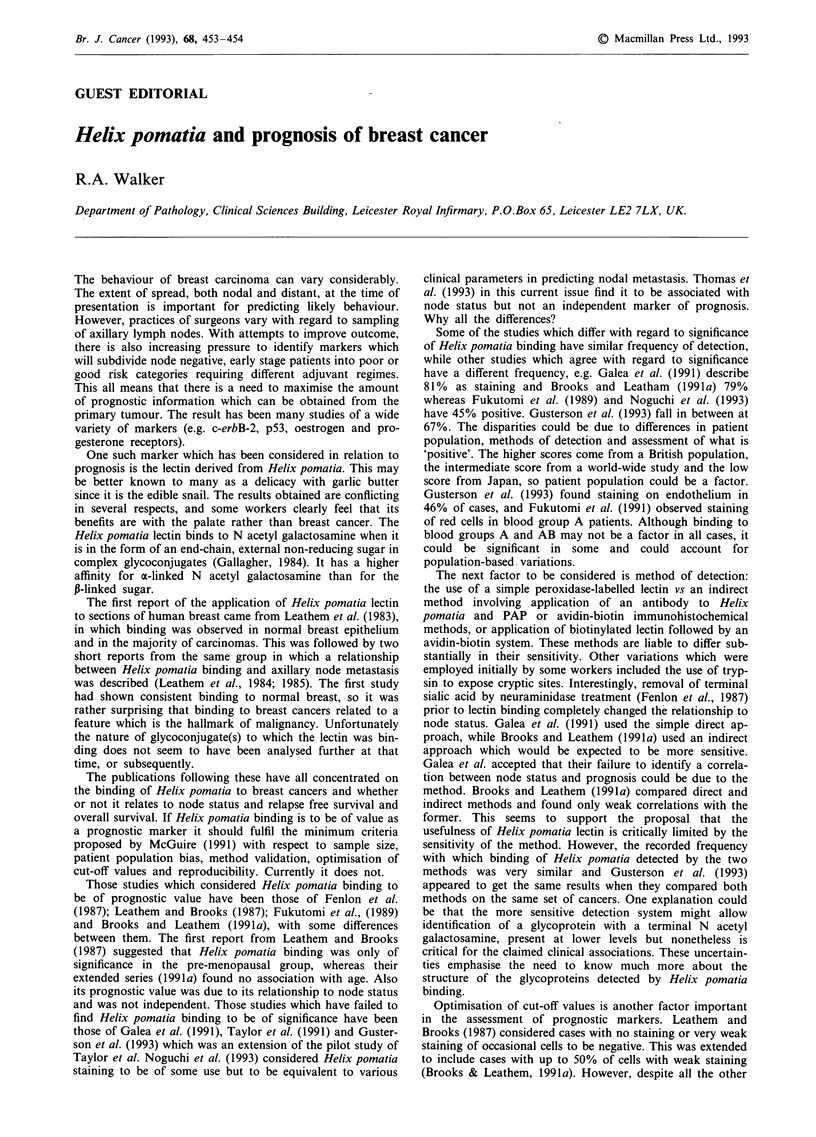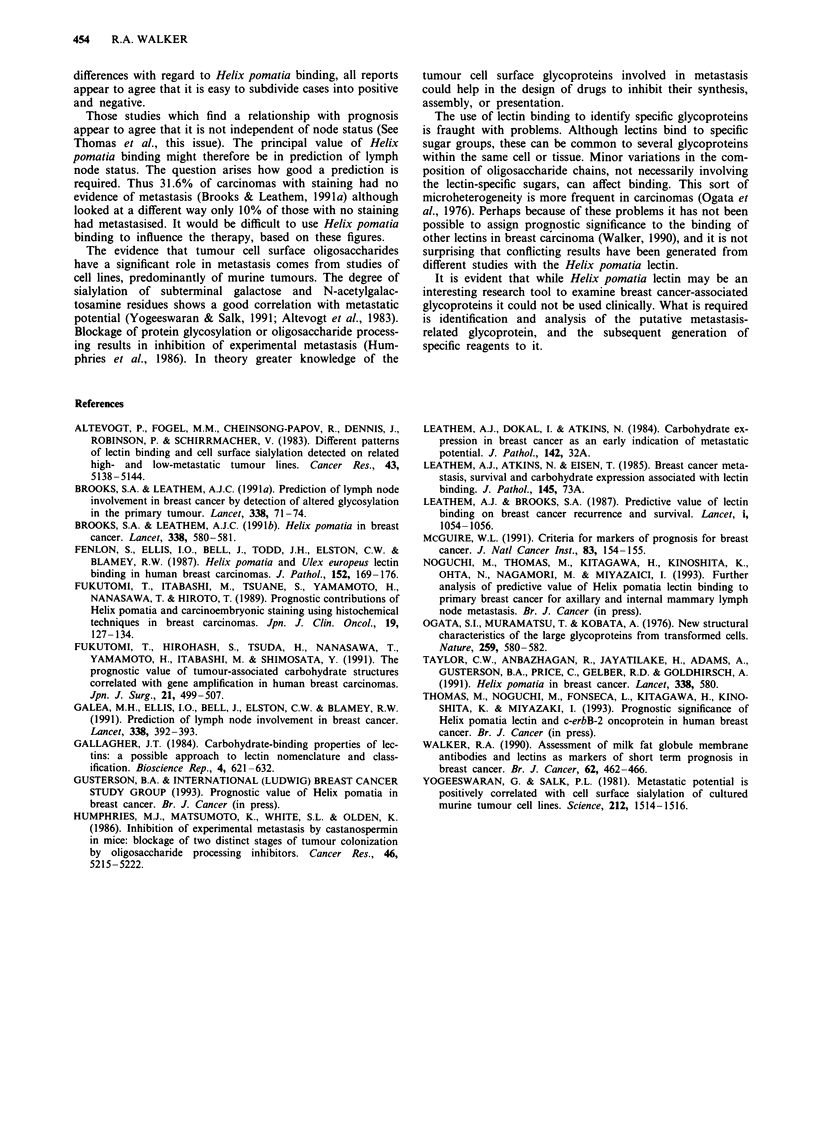# Helix pomatia and prognosis of breast cancer.

**DOI:** 10.1038/bjc.1993.367

**Published:** 1993-09

**Authors:** R. A. Walker


Br. J. Cancer (1993), 68, 453-454 © Macmillan Press Ltd., 1993

**GUEST EDITORIAL**

### *Helix pomatia* and prognosis of breast cancer

R.A. Walker

*Department of Pathology, Clinical Sciences Building, Leicester Royal Infirmary, P.O.Box 65, Leicester LE2 7LX, UK.*

The behaviour of breast carcinoma can vary considerably. The extent of spread, both nodal and distant, at the time of presentation is important for predicting likely behaviour. However, practices of surgeons vary with regard to sampling of axillary lymph nodes. With attempts to improve outcome, there is also increasing pressure to identify markers which will subdivide node negative, early stage patients into poor or good risk categories requiring different adjuvant regimes. This all means that there is a need to maximise the amount of prognostic information which can be obtained from the primary tumour. The result has been many studies of a wide variety of markers (e.g. c-*erb*B-2, p53, oestrogen and progesterone receptors).

One such marker which has been considered in relation to prognosis is the lectin derived from *Helix pomatia*. This may be better known to many as a delicacy with garlic butter since it is the edible snail. The results obtained are conflicting in several respects, and some workers clearly feel that its benefits are with the palate rather than breast cancer. The *Helix pomatia* lectin binds to N acetyl galactosamine when it is in the form of an end-chain, external non-reducing sugar in complex glycoconjugates (Gallagher, 1984). It has a higher affinity for α-linked N acetyl galactosamine than for the β-linked sugar.

The first report of the application of *Helix pomatia* lectin to sections of human breast came from Leathem *et al.* (1983), in which binding was observed in normal breast epithelium and in the majority of carcinomas. This was followed by two short reports from the same group in which a relationship between *Helix pomatia* binding and axillary node metastasis was described (Leathem *et al.*, 1984; 1985). The first study had shown consistent binding to normal breast, so it was rather surprising that binding to breast cancers related to a feature which is the hallmark of malignancy. Unfortunately the nature of glycoconjugate(s) to which the lectin was binding does not seem to have been analysed further at that time, or subsequently.

The publications following these have all concentrated on the binding of *Helix pomatia* to breast cancers and whether or not it relates to node status and relapse free survival and overall survival. If *Helix pomatia* binding is to be of value as a prognostic marker it should fulfil the minimum criteria proposed by McGuire (1991) with respect to sample size, patient population bias, method validation, optimisation of cut-off values and reproducibility. Currently it does not.

Those studies which considered *Helix pomatia* binding to be of prognostic value have been those of Fenlon *et al.* (1987); Leathem and Brooks (1987); Fukutomi *et al.*, (1989) and Brooks and Leathem (1991*a*), with some differences between them. The first report from Leathem and Brooks (1987) suggested that *Helix pomatia* binding was only of significance in the pre-menopausal group, whereas their extended series (1991*a*) found no association with age. Also its prognostic value was due to its relationship to node status and was not independent. Those studies which have failed to find *Helix pomatia* binding to be of significance have been those of Galea *et al.* (1991), Taylor *et al.* (1991) and Gusterson *et al.* (1993) which was an extension of the pilot study of Taylor *et al.* Noguchi *et al.* (1993) considered *Helix pomatia* staining to be of some use but to be equivalent to various clinical parameters in predicting nodal metastasis. Thomas *et al.* (1993) in this current issue find it to be associated with node status but not an independent marker of prognosis. Why all the differences?

Some of the studies which differ with regard to significance of *Helix pomatia* binding have similar frequency of detection, while other studies which agree with regard to significance have a different frequency, e.g. Galea *et al.* (1991) describe 81% as staining and Brooks and Leatham (1991*a*) 79% whereas Fukutomi *et al.* (1989) and Noguchi *et al.* (1993) have 45% positive. Gusterson *et al.* (1993) fall in between at 67%. The disparities could be due to differences in patient population, methods of detection and assessment of what is 'positive'. The higher scores come from a British population, the intermediate score from a world-wide study and the low score from Japan, so patient population could be a factor. Gusterson *et al.* (1993) found staining on endothelium in 46% of cases, and Fukutomi *et al.* (1991) observed staining of red cells in blood group A patients. Although binding to blood groups A and AB may not be a factor in all cases, it could be significant in some and could account for population-based variations.

The next factor to be considered is method of detection: the use of a simple peroxidase-labelled lectin *vs* an indirect method involving application of an antibody to *Helix pomatia* and PAP or avidin-biotin immunohistochemical methods, or application of biotinylated lectin followed by an avidin-biotin system. These methods are liable to differ substantially in their sensitivity. Other variations which were employed initially by some workers included the use of trypsin to expose cryptic sites. Interestingly, removal of terminal sialic acid by neuraminidase treatment (Fenlon *et al.*, 1987) prior to lectin binding completely changed the relationship to node status. Galea *et al.* (1991) used the simple direct approach, while Brooks and Leathem (1991*a*) used an indirect approach which would be expected to be more sensitive. Galea *et al.* accepted that their failure to identify a correlation between node status and prognosis could be due to the method. Brooks and Leathem (1991*a*) compared direct and indirect methods and found only weak correlations with the former. This seems to support the proposal that the usefulness of *Helix pomatia* lectin is critically limited by the sensitivity of the method. However, the recorded frequency with which binding of *Helix pomatia* detected by the two methods was very similar and Gusterson *et al.* (1993) appeared to get the same results when they compared both methods on the same set of cancers. One explanation could be that the more sensitive detection system might allow identification of a glycoprotein with a terminal N acetyl galactosamine, present at lower levels but nonetheless is critical for the claimed clinical associations. These uncertainties emphasise the need to know much more about the structure of the glycoproteins detected by *Helix pomatia* binding.

Optimisation of cut-off values is another factor important in the assessment of prognostic markers. Leathem and Brooks (1987) considered cases with no staining or very weak staining of occasional cells to be negative. This was extended to include cases with up to 50% of cells with weak staining (Brooks & Leathem, 1991*a*). However, despite all the other differences with regard to *Helix pomatia* binding, all reports appear to agree that it is easy to subdivide cases into positive and negative.

Those studies which find a relationship with prognosis appear to agree that it is not independent of node status (See Thomas *et al.*, this issue). The principal value of *Helix pomatia* binding might therefore be in prediction of lymph node status. The question arises how good a prediction is required. Thus 31.6% of carcinomas with staining had no evidence of metastasis (Brooks & Leathem, 1991*a*) although looked at a different way only 10% of those with no staining had metastasised. It would be difficult to use *Helix pomatia* binding to influence the therapy, based on these figures.

The evidence that tumour cell surface oligosaccharides have a significant role in metastasis comes from studies of cell lines, predominantly of murine tumours. The degree of sialylation of subterminal galactose and N-acetylgalactosamine residues shows a good correlation with metastatic potential (Yogeeswaran & Salk, 1991; Altevogt *et al.*, 1983). Blockage of protein glycosylation or oligosaccharide processing results in inhibition of experimental metastasis (Humphries *et al.*, 1986). In theory greater knowledge of the tumour cell surface glycoproteins involved in metastasis could help in the design of drugs to inhibit their synthesis, assembly, or presentation.

The use of lectin binding to identify specific glycoproteins is fraught with problems. Although lectins bind to specific sugar groups, these can be common to several glycoproteins within the same cell or tissue. Minor variations in the composition of oligosaccharide chains, not necessarily involving the lectin-specific sugars, can affect binding. This sort of microheterogeneity is more frequent in carcinomas (Ogata *et al.*, 1976). Perhaps because of these problems it has not been possible to assign prognostic significance to the binding of other lectins in breast carcinoma (Walker, 1990), and it is not surprising that conflicting results have been generated from different studies with the *Helix pomatia* lectin.

It is evident that while *Helix pomatia* lectin may be an interesting research tool to examine breast cancer-associated glycoproteins it could not be used clinically. What is required is identification and analysis of the putative metastasis-related glycoprotein, and the subsequent generation of specific reagents to it.

### References

ALTEVOGT, P., FOGEL, M.M., CHEINSONG-PAPOV, R., DENNIS, J., ROBINSON, P. & SCHIRRMACHER, V. (1983). Different patterns of lectin binding and cell surface sialylation detected on related high- and low-metastatic tumour lines. *Cancer Res.*, **43,** 5138–5144.

BROOKS, S.A. & LEATHEM, A.J.C. (1991*a*). Prediction of lymph node involvement in breast cancer by detection of altered glycosylation in the primary tumour. *Lancet*, **338,** 71–74.

BROOKS, S.A. & LEATHEM, A.J.C. (1991*b*). *Helix pomatia* in breast cancer. *Lancet*, **338,** 580–581.

FENLON, S., ELLIS, I.O., BELL, J., TODD, J.H., ELSTON, C.W. & BLAMEY, R.W. (1987). *Helix pomatia* and *Ulex europeus* lectin binding in human breast carcinomas. *J. Pathol.*, **152,** 169–176.

FUKUTOMI, T., ITABASHI, M., TSUANE, S., YAMAMOTO, H., NANASAWA, T. & HIROTO, T. (1989). Prognostic contributions of Helix pomatia and carcinoembryonic staining using histochemical techniques in breast carcinomas. *Jpn. J. Clin. Oncol.*, **19,** 127–134.

FUKUTOMI, T., HIROHASH, S., TSUDA, H., NANASAWA, T., YAMAMOTO, H., ITABASHI, M. & SHIMOSATA, Y. (1991). The prognostic value of tumour-associated carbohydrate structures correlated with gene amplification in human breast carcinomas. *Jpn. J. Surg.*, **21,** 499–507.

GALEA, M.H., ELLIS, I.O., BELL, J., ELSTON, C.W. & BLAMEY, R.W. (1991). Prediction of lymph node involvement in breast cancer. *Lancet*, **338,** 392–393.

GALLAGHER, J.T. (1984). Carbohydrate-binding properties of lectins: a possible approach to lectin nomenclature and classification. *Bioscience Rep.*, **4,** 621–632.

GUSTERSON, B.A. & INTERNATIONAL (LUDWIG) BREAST CANCER STUDY GROUP (1993). Prognostic value of Helix pomatia in breast cancer. *Br. J. Cancer* (in press).

HUMPHRIES, M.J., MATSUMOTO, K., WHITE, S.L. & OLDEN, K. (1986). Inhibition of experimental metastasis by castanospermin in mice: blockage of two distinct stages of tumour colonization by oligosaccharide processing inhibitors. *Cancer Res.*, **46,** 5215–5222.

LEATHEM, A.J., DOKAL, I. & ATKINS, N. (1984). Carbohydrate expression in breast cancer as an early indication of metastatic potential. *J. Pathol.*, **142,** 32A.

LEATHEM, A.J., ATKINS, N. & EISEN, T. (1985). Breast cancer metastasis, survival and carbohydrate expression associated with lectin binding. *J. Pathol.*, **145,** 73A.

LEATHEM, A.J. & BROOKS, S.A. (1987). Predictive value of lectin binding on breast cancer recurrence and survival. *Lancet*, **i,** 1054–1056.

McGUIRE, W.L. (1991). Criteria for markers of prognosis for breast cancer. *J. Natl Cancer Inst.*, **83,** 154–155.

NOGUCHI, M., THOMAS, M., KITAGAWA, H., KINOSHITA, K., OHTA, N., NAGAMORI, M. & MIYAZAICI, I. (1993). Further analysis of predictive value of Helix pomatia lectin binding to primary breast cancer for axillary and internal mammary lymph node metastasis. *Br. J. Cancer* (in press).

OGATA, S.I., MURAMATSU, T. & KOBATA, A. (1976). New structural characteristics of the large glycoproteins from transformed cells. *Nature*, **259,** 580–582.

TAYLOR, C.W., ANBAZHAGAN, R., JAYATILAKE, H., ADAMS, A., GUSTERSON, B.A., PRICE, C., GELBER, R.D. & GOLDHIRSCH, A. (1991). *Helix pomatia* in breast cancer. *Lancet*, **338,** 580.

THOMAS, M., NOGUCHI, M., FONSECA, L., KITAGAWA, H., KINOSHITA, K. & MIYAZAKI, I. (1993). Prognostic significance of Helix pomatia lectin and c-*erb*B-2 oncoprotein in human breast cancer. *Br. J. Cancer* (in press).

WALKER, R.A. (1990). Assessment of milk fat globule membrane antibodies and lectins as markers of short term prognosis in breast cancer. *Br. J. Cancer*, **62,** 462–466.

YOGEESWARAN, G. & SALK, P.L. (1981). Metastatic potential is positively correlated with cell surface sialylation of cultured murine tumour cell lines. *Science*, **212,** 1514–1516.